# Comprehensive Analysis of the *TaABCB* Gene Family and the Role of *TaABCB7* in the Phosphate Starvation Response in Wheat

**DOI:** 10.3390/biology14111525

**Published:** 2025-10-30

**Authors:** Guoqing Cui, Haigang Wang, Yanzhen Wang, Xia Liu, Menglin Lei, Huibin Qin, Rui Huang, Juan Lu, Zhixin Mu, Yanming Bai

**Affiliations:** 1 Center for Agricultural Genetic Resources Research, Key Laboratory of Crop Gene Resources and Germplasm Enhancement on Loess Plateau, Shanxi Agricultural University, Ministry of Agriculture and Rural Affairs, Taiyuan 030031, China; 2 College of Agriculture, Shanxi Agricultural University, Taigu 030801, China; 3 Yazhouwan National Laboratory, Sanya 572024, China

**Keywords:** genome-wide, *ABCB* subfamily, phosphorus-deficient response, elite haplotype, wheat breeding

## Abstract

**Simple Summary:**

The *ABCB* subfamily is crucial for plant processes like transporting important substances, but its specific role in wheat (*Triticum aestivum* L.) is not well understood. This study found 99 *TaABCB* members in wheat, grouped them into four categories, and analyzed their genome-wide features. Transcriptome analysis showed different expression patterns under phosphate starvation stress, with *TaABCB7* potentially regulating this stress. An elite haplotype H001 of *TaABCB7* was identified and used in wheat breeding. This study deepens our understanding of *TaABCB* members and provides a gene resource for wheat breeding.

**Abstract:**

The *ABCB* subfamily, a subset transporter of the ATP-binding cassette (ABC) superfamily, is vital for various plant life processes, especially in the transport of polar auxin and brassinosteroids. Although ABCB transporters have been characterized in diverse plant species, their specific functions in wheat remain largely unexplored. In this study, we identified 99 *TaABCB* members in wheat and categorized them into four groups based on their conserved domains and phylogenetic relationships. These members were found to be unevenly distributed across all 21 wheat chromosomes. We conducted a comprehensive genome-wide analysis encompassing gene structure, protein motifs, gene duplication events, collinearity, and *cis*-acting elements. Transcriptome analysis revealed that different *TaABCB* members displayed distinct expression patterns under phosphate starvation stress. Notably, we discovered that *TaABCB7* might play a role in regulating wheat’s phosphate starvation. Crucially, we pinpointed an elite haplotype, H001, of the candidate gene *TaABCB7*, which has been progressively selected and employed in wheat breeding improvement programs. Overall, this study enhances our comprehensive understanding of *TaABCB* members and offers a potential gene resource for molecular marker-assisted selection breeding in wheat.

## 1. Introduction

The ATP-binding cassette (ABC) protein superfamily is one of the largest transmembrane transport protein families, widely present in living organisms, including plants, animals, and microorganisms [1,2]. ABC transporters can be classified into three distinct types: full-sized transporters, half-sized transporters, and soluble carriers [3]. The full-sized transporters primarily consist of two highly conserved nucleotide-binding domains (NBDs) and two highly variable transmembrane domains (TMDs). Half-size ABC transporters comprise merely one NBD and one TMD, necessitating the formation of either a homodimer or a heterodimer to execute their functional roles. In contrast, soluble transporters exhibit greater specificity as they encode solely two NBDs without any TMDs [4,5]. NBDs are capable of harnessing energy through the binding and hydrolysis of ATP, thereby facilitating the translocation of diverse substrates across cellular membranes. TMDs are responsible for substrate identification and participate in substrate transmembrane transport [6]. Due to its diversity and complexity, ABC transporters participate in multiple aspects of biological processes in plants, encompassing the maintenance of cellular osmotic balance, the uptake and distribution of plant nutrients, the transport of plant hormones, and signal transduction pathways [7,8].

Based on the phylogenetic analysis of ABC transporter proteins, along with the sequence similarity of nucleotide-binding domains and the structural organization of these domains, the plant ABC transporter protein family can be classified into eight distinct subfamilies: *ABCA*, *ABCB*, *ABCC*, *ABCD*, *ABCE*, *ABCF*, *ABCG*, and *ABCI* [9]. Among these subfamilies, *ABCB* is the second largest subfamily only to the *ABCG* subfamily. In *Arabidopsis thaliana*, a total of 29 *ABCB* transporters have been identified [5], with many of them demonstrated to participate in diverse biological processes, including the transport of phytohormones such as auxin and brassinosteroids [10], stomatal regulation, Fe/S biogenesis and resistance to heavy metal stress (cadmium, lead and aluminum). The most extensive analysis of *ABCB* function has focused on *AtABCB1* (or *AtPGP1*) and *AtABCB19* (or *AtMDR1*/*AtPGP19*). *AtABCB1* and *AtABCB19* are the best characterized members known to function as auxin efflux transporters, which are shared high sequence similarity and clustered together in the phylogenetic tree [11,12]. *AtABCB1* is expressed in both the root and shoot apex, and it colocalizes as well as interacts with TWD1 at specific lateral plasma membrane (PM) domains of epidermal cell layers, thereby facilitating the regulated influx of auxin from the root apoplast into the cytoplasm [13]. *AtABCB19* is predominantly localized within the vascular tissues of the hypocotyl and the stelar region of the root. The loss-of-function mutant of *abcb19* displays a range of auxin-associated phenotypic alterations, while *atabcb1*/*atabcb19* double knockout plants exhibit even more dramatic phenotypes, encompassing dwarfism, diminished apical dominance in mature plants, a shortened hypocotyl under both light and dark conditions, as well as abbreviated stamens [14,15,16]. Interestingly, the latest research has found that *ABCB19* also could act as a brassinosteroid exporter and positively regulates brassinosteroid signaling together with *ABCB1* [17]. However, *AtABCB4*, homolog to *AtABCB21* [18,19], exhibited auxin concentration-dependent influx/efflux transporter activity from the root tip to the root elongation zone, when the level of auxin in cells is low, it promotes auxin input, and when it is high, it promotes auxin output [20]. Loss-of-function mutants of *AtABCB4* exhibited an abnormal gravitropic response, irregular lateral root development, and increased root hair elongation [12]. When *ABCB4* is overexpressed under the regulation of a root hair-specific promoter, it leads to shorter root hairs, a phenotype reminiscent of that observed with the overexpression of PIN efflux transporters [21]. Additionally, *AtABCB14* regulates stomatal movement in response to CO_2_ by facilitating the transport of malate from the apoplast into guard cells, which subsequently elevates their osmotic pressure [22]. Meanwhile, *OsABCB14* functions as an auxin influx transporter that plays a role in iron (Fe) homeostasis [23]. *ABCB8* is crucial for maintaining mitochondrial iron homeostasis and the maturation of cytosolic Fe/S proteins, while the mitochondrial ABC transporter *AtABCB25* contributes to Fe/S cluster formation by exporting glutathione polysulfide from the mitochondria to facilitate cytosolic Fe/S cluster assembly [24,25]. Moreover, *ABCB25* also confers tolerance to heavy metals such as cadmium, lead, and aluminum [26,27]. Furthermore, *AtABCB27* (*AtTAP2*/*ALS1*) and *OsABCB27* have been implicated in aluminum tolerance [28,29,30].

Phosphate is an essential macronutrient for plant growth and development, playing crucial roles in various physiological processes such as energy metabolism, nucleic acid synthesis, and signal transduction. However, phosphate deficiency is a common abiotic stress that significantly limits crop yield and quality worldwide. Plants have evolved a series of complex adaptive mechanisms to cope with phosphate starvation, including changes in root architecture, secretion of organic acids and phosphatases, and alteration of gene expression patterns. Given that ABCB transporters are broadly involved in the uptake and distribution of plant nutrients, it is highly likely that members of the ABCB subfamily participate in the plant’s response to phosphate starvation. For instance, they may be involved in the transport of phosphate or phosphate—related compounds across cellular membranes, or in the regulation of signaling pathways that respond to phosphate deficiency.

However, a comprehensive analysis regarding the identification and functional characterization of the *TaABCB* gene family in wheat (*Triticum aestivum* L.) has not been reported. In this study, we conducted a genome-wide bioinformatics analysis to identify the numbers, physicochemical properties of proteins, and subcellular localization of *TaABCB* gene family members. Subsequently, we delved into the phylogenetic relationships, gene structure, conserved motifs, chromosomal distribution, gene duplication events, collinearity patterns, and *cis*-acting elements associated with these family members. We also performed transcriptome analysis to investigate the potential roles of *TaABCB* members in response to phosphate starvation. By integrating low-phosphorus transcriptome data with RT-qPCR analysis of candidate genes, we identified *TaABCB7* as a potential key gene involved in the response to phosphorus deficiency stress. In addition, we discovered an elite haplotype of *TaABCB7*. These findings offer valuable insights for further research into the molecular functions of *TaABCB* gene family members and provide a potentially useful gene for molecular marker-assisted selection breeding in wheat.

## 2. Materials and Methods

### 2.1. Identification and Physicochemical Properties Analysis of TaABCB Members

The genomic data of wheat (Chinese Spring cultivar), encompassing the reference genome sequence, protein sequences, and gff3 files, were sourced from the Ensembl Plants database (https://plants.ensembl.org/index.html, accessed on 10 June 2024). Protein sequences of 29 *AtABCB* members in *Arabidopsis thaliana* (Supplementary Table S1) were retrieved from the National Center for Biotechnology Information database (https://www.ncbi.nlm.nih.gov/, accessed on 10 June 2024) and utilized as query templates in the BLASTP tool within TBtools-II (version 2.313) software [31] to identify all potential *TaABCB* members. Hidden Markov Models (HMMs) for the conserved domains (PF00005 and PF00664) of *AtABCB* were downloaded from the Pfam database (http://pfam.xfam.org/, accessed on 11 June 2024). The HMMER tool [32] was then employed to search and compare the entire genome’s protein sequences against these two *AtABCB* transporter HMMs. Additionally, *TaABCB* members were validated using the NCBI-CDD tool (https://www.ncbi.nlm.nih.gov/Structure/bwrpsb/bwrpsb.cgi, accessed on 11 June 2024) [33,34,35]. By integrating the results from BLASTP, HMMER, and NCBI-CDD, the members of the *TaABCB* gene family in wheat were further confirmed using the Triticeae-GeneTribe (http://wheat.cau.edu.cn/TGT/, accessed on 12 June 2024) with functional annotations [36].

Subsequently, the protein sequences of the selected members were submitted to the protein parameter calculator in TBtools-II (version 2.313) to predict the following physicochemical properties: amino acid count, molecular weight (MW), theoretical isoelectric point (pI), instability index, aliphatic index, and grand average of hydropathicity (GRAVY) values. The subcellular localization of TaABCBs was predicted using WOLF PSORT (http://wolfpsort.hgc.jp/, accessed on 15 June 2024), Busca (http://busca.biocomp.unibo.it/, accessed on 15 June 2024) [37], and Plant-mPLoc (http://www.csbio.sjtu.edu.cn/bioinf/plant-multi/, accessed on 15 June 2024) [38].

### 2.2. Multiple Sequence Alignment and Phylogenetic Tree Construction

To elucidate the evolutionary relationships among the members, a total of 99 identified *TaABCBs*, along with 27 *OsABCBs* [39] and 29 *AtABCBs* protein sequences, were aligned using ClustalW in MEGA 12 (version 12.0.11) software [40]. The multiple sequence alignment parameters in the ClustalW Parameters window are configured as follows: For pairwise alignment, the Gap Opening Penalty is set to 10.00 and the Gap Extension Penalty to 0.10. For Multiple Alignment, the Gap Opening Penalty is 10.00 and the Gap Extension Penalty is 0.20. In the Matrix section, the Protein Weight Matrix is Gonnet, Residue-specific Penalties and Hydrophilic Penalties are both ON, the Gap Separation Distance is 4, the End Gap Separation and the Use Negative Matrix are OFF, the Delay Divergent Cutoff is 30%.

The maximum likelihood (ML) method in MEGA 12 (version 12.0.11) software was employed to construct a phylogenetic tree. To ensure statistical reliability, the analysis incorporated 1000 bootstrap replicates. The Jones–Taylor–Thornton (JTT) substitution model was selected due to its appropriateness for the amino acid composition of transmembrane transporters. To address evolutionary rate variation, a uniform rate was enforced across all alignment positions, while gaps and missing data were retained in the dataset to maintain sequence integrity. The tree topology was optimized using the nearest-neighbor-interchange (NNI) heuristic algorithm, with parallel processing (eight threads) employed to improve computational efficiency. The resulting phylogenetic tree was refined and visualized using the online iTOL (http://itol.embl.de/, accessed on 12 May 2025) [41], where distinct clade-specific color coding was applied to facilitate cross-species comparative analysis and subgroup identification.

### 2.3. Characterization of Conserved Motifs and Domains of TaABCB Members

The conserved motifs of *TaABCB* members were identified using the Multiple Expectation maximization for Motif Elicitation (MEME) database (https://web.mit.edu/meme/current/share/doc/meme.html, accessed on 20 June 2024). The maximum number of motifs was set to 10, *p*-value < 0.01, and other parameters were set at the default values. The conserved domains of *TaABCB* members were predicted using the Batch CD Search tool on NCBI (https://www.ncbi.nlm.nih.gov/Structure/bwrpsb/bwrpsb.cgi, accessed on 8 May 2025). TBtools-II (version 2.313) was employed for visualizing both the gene structure and motifs composition.

### 2.4. Chromosomal Mapping, Gene Duplication, and Collinearity Analysis of TaABCB Members

The chromosome mapping of *TaABCB* members was constructed using TBtools-II (version 2.313) according to the positional information of these members retrieved from the reference gff3 files. Gene duplication events of *TaABCB* members were analyzed using MCScanX tool and then visualized using Advanced Circos tool in TBtools-II (version 2.313). To investigate the selection pressure of *TaABCB* members during evolution, the KaKs Calculator tool was employed to calculate the nonsynonymous substitution (Ka) and synonymous substitution (Ks) rates of duplicated genes in the *TaABCBs* gene family [42]. In addition, collinearity analysis of *TaABCB* members was performed using MCScanX tool in wheat and other species (*Zea mays B73*, *Oryza sativa Japonica* and *Arabidopsis thaliana thale cress*). The genome gff3 files, genome sequences, and protein sequence files of maize, rice, and *Arabidopsis thaliana* were downloaded from the Ensembl Plants database (https://plants.ensembl.org/index.html, accessed on 10 June 2024), and then use the Dual Synteny Plot tool in TBtools-II (version 2.313) for plotting.

### 2.5. Cis-Acting Elements Prediction and Gene Structure Analysis

For the analysis of *cis*-acting elements, promoter sequences of *TaABCB* gene family members, encompassing the 2000 bp region upstream of the initiation codon “ATG” were extracted from the wheat reference genome sequences. Subsequently, the *cis*-acting elements within these promoter regions were predicted using the PlantCARE database (https://bioinformatics.psb.ugent.be/webtools/plantcare/html/, accessed on 30 June 2024). Elements associated with plant growth and development, phytohormone responsiveness, as well as biotic and abiotic stress responses, were selected for detailed analysis. Distribution maps illustrating the *cis*-acting elements of *TaABCB* members were generated utilizing TBtools-II (version 2.313). Additionally, the gene structure of *TaABCB* members, including the positions and numbers of exons and introns, was analyzed based on gff3 files using the Gene Structure View (Advanced) feature within TBtools-II (version 2.313) software.

### 2.6. RNA-Seq Analysis

For RNA-seq analysis, the study focused on an elite bread wheat cultivar, Kenong199 (KN199). KN199 seeds were germinated and cultivated in nutrient solutions composed of 1.5 mM CaCl_2_, 1 mM MgSO_4_, 10 μM H_3_BO_3_, 1 μM ZnSO_4_·7H_2_O, 0.5 μM CuSO_4_·5H_2_O, 2 mM Ca(NO_3_)_2_·4H_2_O, 0.1 mM FeNaEDTA, 1 μM MnSO_4_·H_2_O, 1.5 mM KCl, 0.05 μM (NH_4_)_6_Mo_7_O_24_·4H_2_O, 0.2 mM NaH_2_PO_4_. The pH of the nutrient solution was adjusted to 6.0 using HCl or NaOH, and the solution was refreshed every three days. Two-week-old wheat seedlings, which had developed three leaves and were at the autotrophic stage with heightened sensitivity to abiotic stresses [43], were subjected to either phosphorus-sufficient (0.2 mM, PS) or phosphorus-deficient (0 mM, PD) conditions, while maintaining identical concentrations of all other nutrients in both growth media. All seedlings were grown in a controlled growth chamber at 25 °C with a 16 h light and 18 °C with 8 h dark cycle, and a relative humidity of approximately 70%.

Root samples (three biological replicates) were collected after one day of treatment. Total RNAs were extracted using ethanol precipitation and CTAB-PBIOZOL methods. The quality and quantity of total RNA were assessed using a Qubit 4.0 fluorescence quantifier (Thermo Fisher Scientific, Waltham, MA, USA) and a Qsep400 high-throughput biofragment analyzer (Advanced Instruments, Inc., Norwood, USA), respectively. The preparation of RNA-seq libraries and subsequent sequencing were carried out by Metware Biotechnology Co., Ltd. (Wuhan, China). Briefly, transcriptome libraries were constructed by enriching mRNA with polyA tails using Oligo (dT) magnetic beads, followed by sequencing on the Illumina Hiseq platform (Illumina, Inc., San Diego, CA, USA) after passing library quality checks.

After filtering out low-quality reads from the raw sequence data using fastp (version 0.24.0) [44], the clean reads were aligned to the wheat reference genome (International Wheat Genome Sequencing Consortium, RefSeq v2.0) using HISAT2 [45]. For differential expression analysis, we used raw counts as input for DESeq2 [46,47]. This aligns with DESeq2’s requirement for discrete count data to accurately model gene expression dispersion and normalize for sequencing depth. FPKM (fragments per kilobase of transcript per million fragments mapped) values were computed for visualizing expression patterns. FPKM normalizes for both gene length and sequencing depth, making it suitable for relative expression comparison of the same gene across samples, with a false discovery rate (FDR) threshold of <0.05 and |log_2_ Fold Change| ≥ 0.6.

### 2.7. RNA Extraction and RT-qPCR Analysis

For RT-qPCR analysis, two-week-old KN199 wheat seedlings were subjected to treatment under Pi-sufficient (0.2 mM, PS) and Pi-deficient (0 mM, PD) conditions for 1, 3, and 5 days, respectively. Subsequently, total RNA was extracted from the young roots of these seedlings using the TRIzol reagent (Invitrogen, Carlsbad, CA, USA) according to the manufacturer’s instructions. For the quality and purity evaluation of the extracted RNA, absorbance values at 260 nm and 280 nm were determined with a NanoDrop spectrophotometer (Thermo Fisher Scientific, Waltham, MA, USA), while RNA integrity was verified via electrophoresis on a 1.2% agarose gel. Full-length cDNA was synthesized using the 5× All-In-One RT Master Mix system (Abm Inc., New York, NY, USA). RT-qPCR analysis was performed using a LIGHTCYCLER 96 (Roche, Basel, Switzerland), with each assay replicated at least three times using three independent RNA preparations. *TaActin* transcripts were used as an internal reference for normalization. Gene expression levels were calculated using the 2^−ΔΔCT^ method [48], where CT represents the threshold cycle. Primer sequences are provided in Supplementary Table S6.

### 2.8. Computational Analysis of AlphaFold 3

To predict the conjunction of TaPHR2 and the promoter of *TaABCB7*, AlphaFold 3 was employed for conformational prediction analysis, and yielded a PDB file with a pTM and ipTM value. The value of pTM + ipTM ≥ 0.75 indicates a good docking effect and reliable results.

For the purpose of molecular docking analysis, the PDB file of TaPHR2 was input as the ligand, while the PDB file of the *TaABCB7* promoter served as the receptor into the HDOCK server. The docking conformations were generated using HDOCK with a grid spacing parameter set to 1.2 and an angle parameter set to 15. The docking score was calculated using the formula: Confidence score (CS) = 1.0/[1.0 + e^0.02×(Docking_Score+150)^]. The confidence score, based on a docking score of −200 (where a score less than −200 indicates a high probability of protein interaction), is approximately 0.7. CS ≥ 0.7, it indicates that there is a high probability of binding between the two molecules; 0.7 > CS ≥ 0.5, it indicates that the two molecules may be bound; 0.5 > CS, it indicates that the two molecules are unlikely to combine.

### 2.9. Dual-Luciferase Reporter Assays

The promoter region of *TaABCB7* (approximately 2000 bp in length) was amplified from the genomic DNA of KN199 and subsequently inserted into the *pGreenII0800* vector to create the reporter construct *35S::proTaABCB7-LUC-35S::REN*. Simultaneously, the full-length coding sequence of *TaPHR2* was amplified from the cDNA of KN199 and inserted into the *pGreenII 62-SK* overexpression vector to generate the effector construct *35S::TaPHR2*. Both constructs were co-transfected into *Nicotiana benthamiana* leaf protoplasts using PEG-mediated transformation. After transfection (16–18 h later), the activities of firefly luciferase (LUC) and Renilla luciferase (REN) were measured using the Dual-Luciferase^®^ Reporter Assay System (Promega, E1960, Madison, WI, USA) on a microplate reader. The LUC activity was normalized to the REN activity to account for variations in transfection efficiency. The data are presented as mean ± *SD* (*n* = 4). Statistical significance was assessed using Student’s *t*-test (* *p* ≤ 0.05, ** *p* ≤ 0.01, *** *p* ≤ 0.001). Primer sequences are listed in Supplementary Table S6.

### 2.10. The Haplotype Analysis of TaABCB7

To investigate the selection of the *TaABCB7* in breeding and improvement processes, the *TaABCB7* gene resequencing data were extracted from 406 wheat genotypes in the Wheat Genotype and Phenotype Database (http://resource.iwheat.net/WGPD/, accessed on 30 June 2024) [49] for haplotype classification, and the geneHapR package (version 1.2.4) was used for haplotype analysis. Then, significant differences in plant height (PH), productive tiller number (PTN), thousand kernel weight (TKW) and total root length (TRL) were analyzed among different haplotypes.

## 3. Results

### 3.1. Identification of TaABCB Gene Family Members in Wheat

In this study, a total of 99 *TaABCB* members were identified from wheat genome (Chinese Spring cultivar) and systematically named according to their chromosomal locations (detailed characteristics of these members were listed in Supplementary Table S2). The encoded proteins exhibited notable variability in length, ranging from 488 amino acids (aa) in *TaABCB11* to 1500 aa in *TaABCB68*, with corresponding molecular weights spanning 53,286.86 Da (*TaABCB11*) to 160,898.77 Da (*TaABCB68*). The predicted isoelectric points (pI) varied from 5.71 (*TaABCB89*) to 9.38 (*TaABCB91*). Detailed analysis of the physicochemical properties revealed instability indices ranging from 26.22 to 46.32, aliphatic indices between 89.34 and 107.95, and grand average of hydropathicity (GRAVY) scores from −0.056 to 0.206. Subcellular localization predictions indicated that all TaABCB proteins are membrane-bound, suggesting their potential regulatory functions in cellular membrane processes.

### 3.2. Phylogenetic Analysis of TaABCB Members

**Figure 1:**
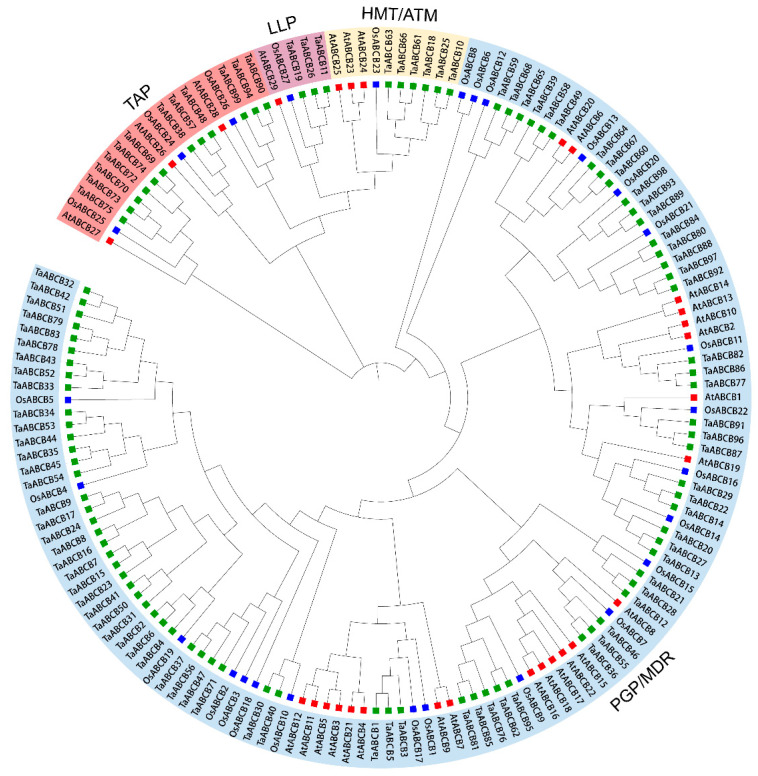
Phylogenetic analyses were conducted on ABCB proteins from *Arabidopsis thaliana*, rice (*Oryza sativa Japonica*), and wheat (*Triticum aestivum* L.). Different colored boxes represent different species: *Arabidopsis thaliana* (red), *Oryza sativa* (blue), and *Triticum aestivum* (green). Abbreviations: PGP/MDR (P-glycoprotein multidrug resistance); TAP (Transporter associated with antigen processing); HMT/ATM (Heavy metal tolerance/ABC transporters of the mitochondrion); LLP (Prokaryotic lipid A-like exporters, putative).

To explore the evolutionary relationships among *TaABCB* family members, a phylogenetic tree was constructed using full-length protein sequences from *Arabidopsis thaliana*, rice, and wheat as reference datasets. Based on the *AtABCB* subfamily has been divided into two major classes: full-size transporters (only include PGP/MDR type) and half-size transporters (include LLP, HMT/ATM, and TAP types). Using subpopulation division of *Arabidopsis thaliana* as a reference, 99 *TaABCB* members were divided into four branches. Among them, 78 *TaABCB* members were clustered into the MDR type transporters, 3 *TaABCB* members belong to the LLP type transporters, 6 *TaABCB* members belong to the ATM type transporters, and 12 *TaABCB* members belong to the TAP type transporters (Figure 1).

### 3.3. Conserved Motifs and Domains Analysis of TaABCB Members

**Figure 2:**
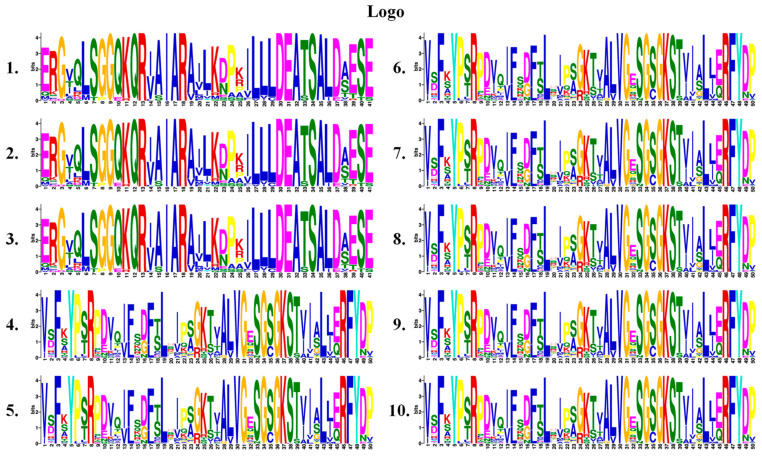
Conservative motifs logo of *TaABCBs* members in wheat. The conserved motifs within the amino acid sequences of TaABCB members have been identified.

**Figure 3:**
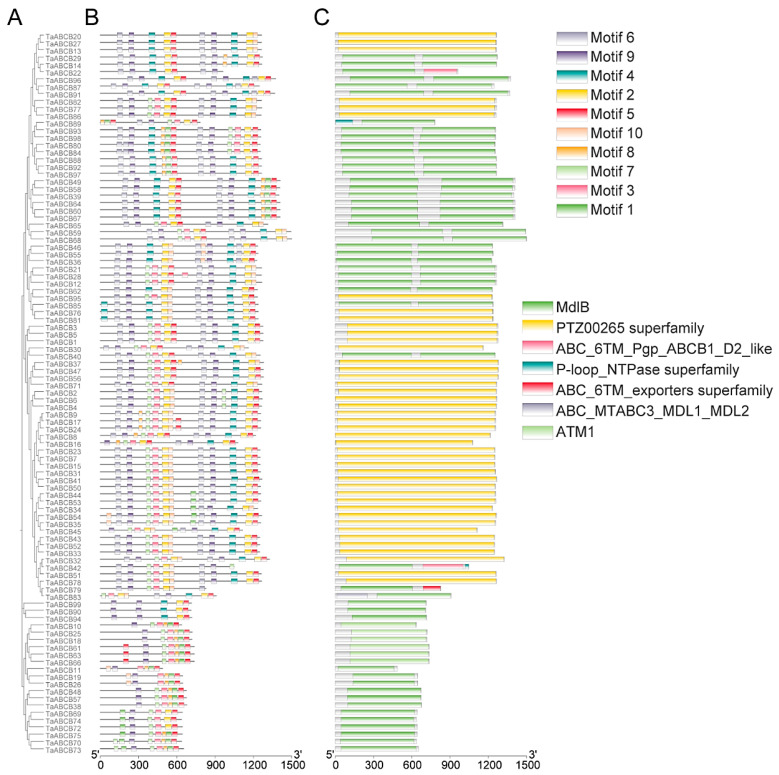
Phylogenetic tree, conserved motifs, and domains analyses of *TaABCBs* members in wheat. (**A**) The phylogenetic tree of the 99 *TaABCB* members. (**B**) Conserved motifs distribution of *TaABCB* members. (**C**) Domains distribution of *TaABCB* members.

To elucidate the structural diversity among *TaABCB* family members, conserved motifs and functional domains were systematically mapped based on genomic annotation data. Firstly, an evolutionary tree was constructed for the *TaABCB* members using MEGA software and categorized them into four types: PGP/MDR, HMT/ATM, TAP, and LLP. We then analyzed the motif patterns in *TaABCB* members to further elucidate the evolutionary relationships. A total of 10 conserved motifs were detected across the 99 *TaABCB* members, designated as motif 1 to motif 10 (Figure 2). According to the structural features of ABCB proteins based on evolutionary tree (Figure 3A), the 78 full-size TaABCB proteins in Group MDR included 7 to 13 motifs, of which 8 motifs (2, 3, 4, 5, 6, 7, 9 and 10) being highly prevalent and constituting core components of their sequences. The 21 half-size TaABCB proteins contained 5 to 8 motifs and 6 motifs (1, 3, 5, 7, 8 and 9) were highly conserved, for instance, 6 ATM type of half-size transporters possessed 6 or 7 motifs, 12 TAP type transporters contained 5–8 motifs, and 3 LLP type transporters contained 6 motifs, respectively (Figure 3B). All TaABCB family members share the ABC_STABC3_SDL1_MDL2 domains, with members of the same subgroup displaying similar domain architectures and motif counts (Figure 3C). Conserved motifs and domains analysis showed that most TaABCB proteins tended to have similar conserved motifs and domains compositions in separate subgroups. These results are consistent with the evolutionary pattern presented by the phylogenetic tree, indicating that functional divergence likely occurred among subgroup members during *TaABCB* evolution.

### 3.4. Chromosomal Mapping, Gene Duplication, and Collinearity Analysis

**Figure 4:**
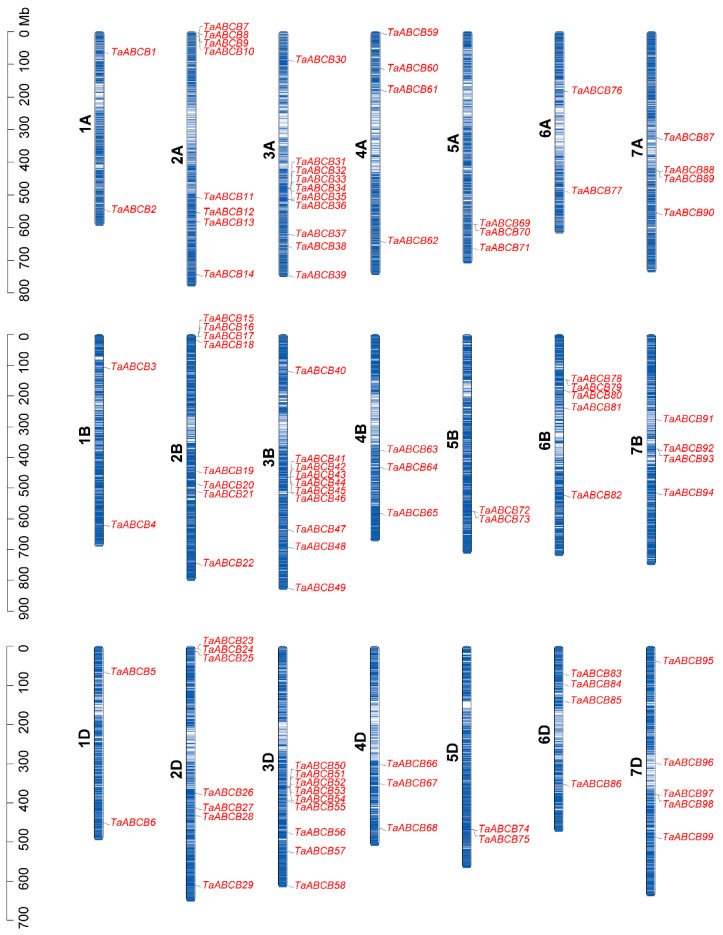
Distribution of *TaABCB* genes across wheat chromosomes, with the left-hand scale in megabases (Mb).

To determine the distribution of the *TaABCB* members, we analyzed their distribution across the wheat chromosomes. Chromosome mapping analysis showed that the 99 *TaABCB* members had an unevenly distribution on all 21 chromosomes (Figure 4). Among these chromosomes, Chr3 (with 29 *TaABCBs*) and Chr2 (with 23 *TaABCBs*) harbored the highest density of *TaABCB* members. 3A and 3B each contain 10, 3D contains 9, 2A and 2B each contain 8, and 2D contains 7, followed by Chr7 with 13 *TaABCBs*, Chr 6 with 11 *TaABCBs*, and Chr4 with 10 *TaABCBs*, respectively. Chr5 with 7 *TaABCBs* and Chr1 with 6 *TaABCBs* possessed the least.

**Figure 5:**
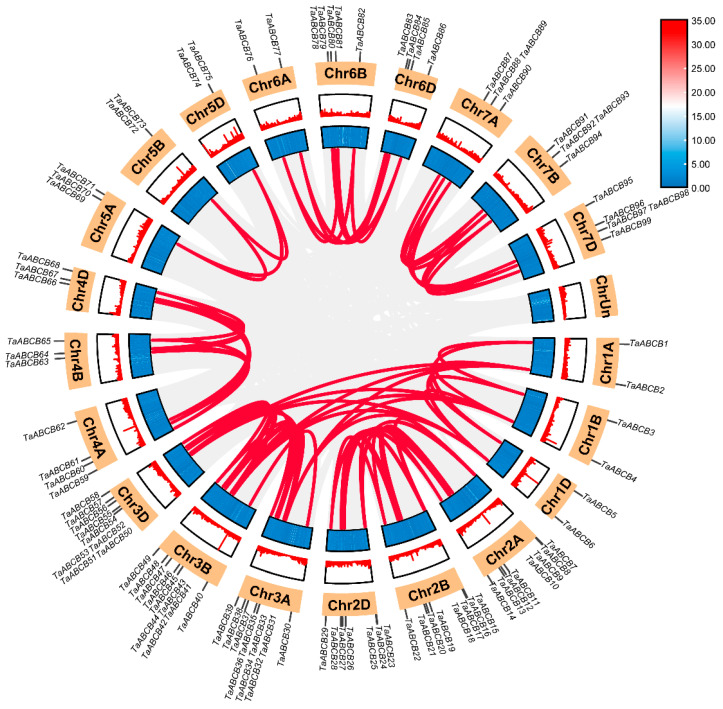
Collinearity analysis of *TaABCB* gene family members in wheat. The collinear gene pairs are marked by red lines. The red-to-blue gradient regions on the chromosomes represent gene density (red indicates dense gene distribution, while blue indicates sparse distribution), and the color scale on the right clarifies the density value range (0.00–35.00).

Gene duplication serves as the primary driver of gene family evolution, facilitating the rapid expansion of member numbers and functional diversification. This process has been critical for wheat’s adaptation to diverse environmental conditions during polyploidization, primarily through segmental and tandem duplication mechanisms. To gain deeper insights into the dynamics of gene duplication events in wheat, we analyzed the segmental and tandem duplications that occurred among 99 *TaABCB* members. Herein, a total of 79 segmental duplication gene pairs were identified across the TaABCB family, distributed on distinct chromosomes, with no tandem duplication events detected. Meanwhile, 9 segmental duplication pairs were found between homologous chromosome groups 1A/1B/1D and 3A/3B/3D. These results indicated that segmental duplication has been the dominant mechanism driving TaABCB gene family expansion, particularly during wheat polyploidization (Figure 5). Furthermore, Ka/Ks ratio analysis revealed that all collinear gene pairs exhibited values less than 1, indicating that these genes have undergone purifying selection throughout their evolutionary process (Supplementary Table S3).

To investigate the evolutionary origin and homology of 99 *TaABCB* members, a collinearity analysis was performed through comparing wheat with three species, including two monocotyledons (*O. sativa* and *Z. mays*) and a dicotyledons *Arabidopsis thaliana*. The results showed that 7, 56 and 66 pairs of homologous genes from *Arabidopsis thaliana*, rice and maize, respectively.

**Figure 6:**
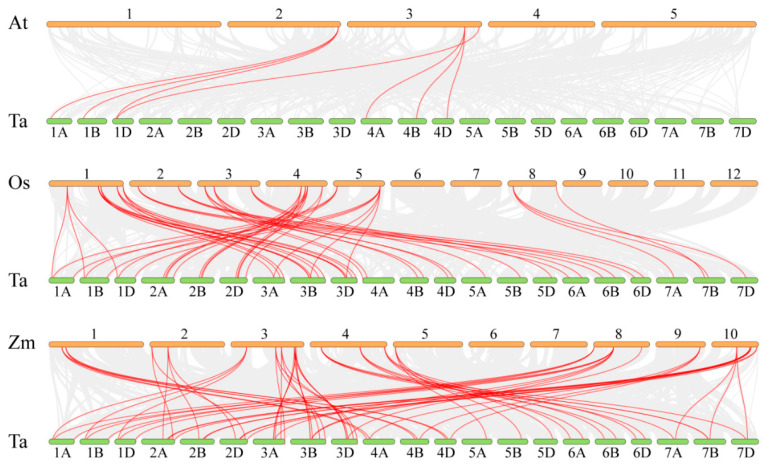
Collinearity analyses of *ABCB* members between wheat and other species. The red lines represent collinear gene pairs between species, Ta represents *Triticum aestivum* (wheat), At represents *Arabidopsis thaliana*, Os represents *Oryza sativa* (rice), and Zm represents *Zea mays* (maize).

This suggests that *TaABCB* genes exhibit a closer evolutionary relationship with their orthologs in monocotyledonous species such as rice and maize. Compared to other species, wheat undergoes rapid gene expansion during the process of polyploidization, and the *TaABCBs* may have played a critical role in shaping wheat’s evolutionary trajectory (Figure 6).

### 3.5. Cis-Acting Elements and Gene Structure Analysis of TaABCB Members

**Figure 7:**
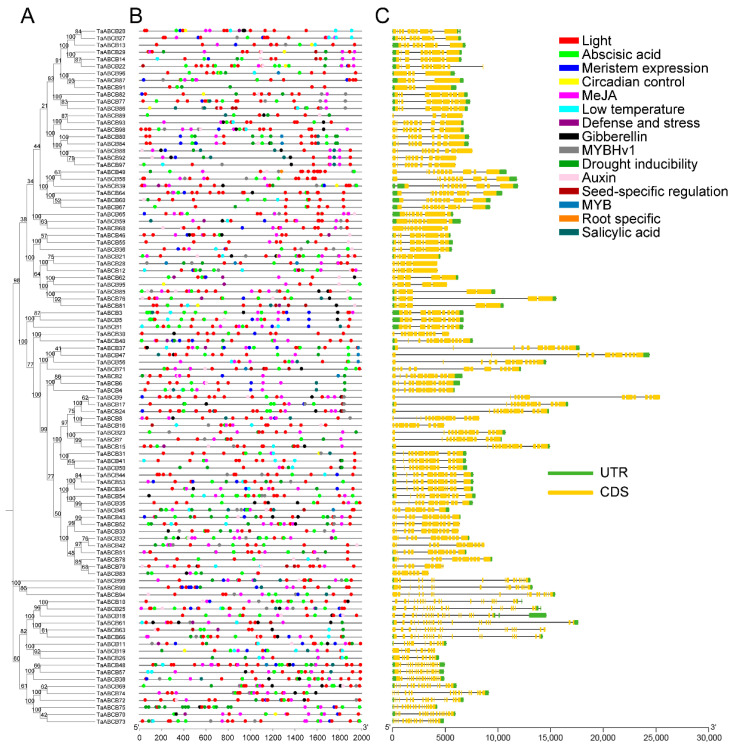
*Cis*-acting elements and gene structure analysis of *TaABCB* members. (**A**) Evolutionary tree of *TaABCBs*. (**B**) Promoter *cis*-regulatory element distribution in *TaABCB* genes. (**C**) Gene structure analysis of *TaABCBs* members.

Gene expression is predominantly regulated by *cis*-acting elements in promoter regions located upstream of the transcription start site. To elucidate the expression patterns and potential regulatory roles of *TaABCB* genes, we analyzed the promoter sequences of 99 *TaABCB* members using the PlantCARE database. This revealed diverse functional *cis*-elements associated with plant growth and development, phytohormone signaling, and biotic/abiotic stress responses (Figure 7A,B). Key growth-related elements included the circadian element (circadian regulation), RY-element (seed-specific expression), CAT-box (meristem activity), motif I (root-specific expression), and light-responsive motifs (ACE, G-Box, Sp1, GT1-motif, TCCC-motif), suggesting multifaceted roles for TaABCBs in plant. Promoters also contained multiple phytohormone-responsive elements, such as auxin-related (TGA-element, AuxRR-core), gibberellin-related (TATC-box, P-box, GARE-motif), abscisic acid-responsive (ABRE), methyl jasmonate-responsive (CGTCA-motif, TGACG-motif), and salicylic acid-responsive (TCA-element, SARE), indicating that *TaABCB* expression may be modulated by these hormones to influence growth and development. Additionally, stress-related elements like TC-rich repeats (defense signaling) and LTR (low-temperature response) were identified, implying roles in biotic and abiotic stress adaptation. Interestingly, we also discovered four MYB binding related components, including MBS, MBSI, MRE and CCAAT-box. Past studies have shown that MYB transcription factors (AtPHR1 or OsPHR2) can participate in phosphorus starvation response, suggesting that *TaABCB* members may also participate in phosphate deficiency signaling.

Regarding gene structure, *TaABCB* members exhibited exon counts ranging from 4 to 20, in which 78 MDR type of full-size transporters contained 4–13 exons, while half-size transporters had similar exon numbers, of which 6 ATM type of half-size transporters possessed 20 exons, 12 TAP type transporters contained 16–18 exons, and 3 LLP type transporters contained 10 and 11 exons. This structural diversity underscores a complex evolutionary process for the *TaABCB* family in wheat (Figure 7C).

### 3.6. Phosphorus Starvation Transcriptome Analysis

**Figure 8:**
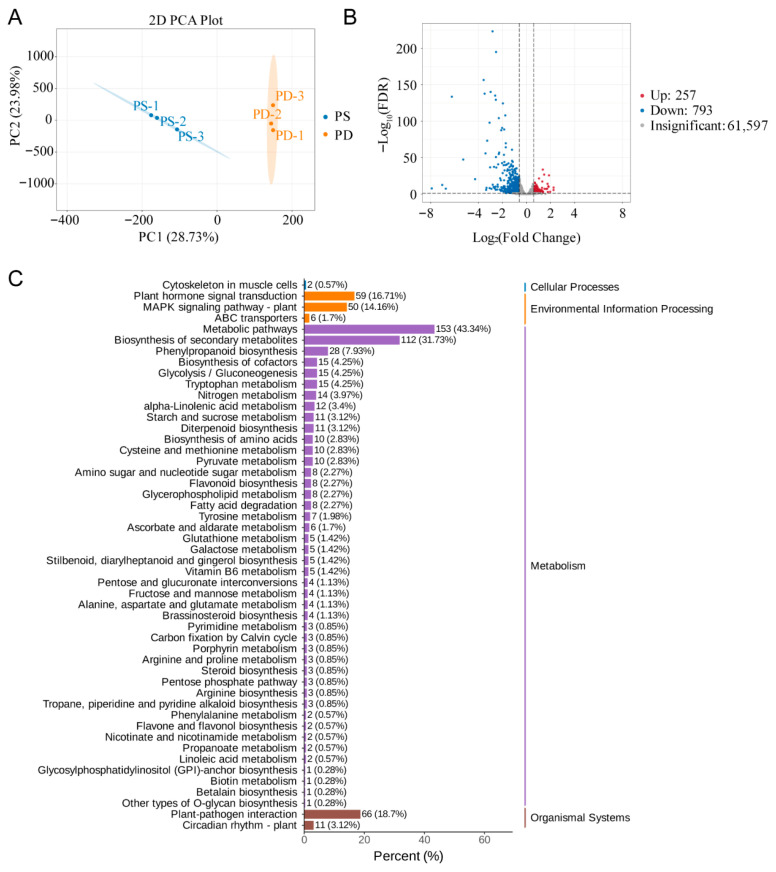
Transcriptomic analysis of Kenong199 under low phosphorus conditions. (**A**) PCA. PS, Phosphorus-sufficient treatment; PD, Phosphorus-deficient treatment. (**B**) Volcano diagram of differentially expressed genes in low phosphorus transcriptome. (**C**) KEGG analysis of differentially expressed genes.

To assess how TaABCB proteins function in the early phase of phosphate starvation response, we sequenced the transcriptome of the root under Pi-sufficient (PS) and Pi-deficiency (PD). The transcriptome sequencing generated 596,956,818 raw reads. Following rigorous quality control to filter low-quality and adapter-contaminated sequences, a total of 563,793,202 high-quality clean reads were retained across six samples, yielding 84.57 Gb of transcriptomic data. Quality metrics showed exceptional consistency, with Q30 bases ranging from 95.99% to 96.13% and an average GC content of 52.75%. Alignment analysis using a sliding window density approach demonstrated high genomic mapping efficiency, with 87.13% of the generated reads were accurately aligned to the wheat reference genome (Chinese Spring cultivar). Notably, over 81% of transcripts exhibited unique mappings, underscoring the robustness and specificity of the alignment process (Supplementary Table S4). Principal component analysis (PCA) demonstrated that PC1 and PC2 accounted for 28.73% and 23.98% of the total variance, respectively, enabling clear differentiation between treatment conditions. Biological replicates clustered tightly in the ordination space, indicating high reproducibility (Figure 8A). Transcriptomic profiling under PS and PD conditions revealed 1050 differentially expressed genes (DEGs) in root tissues, with 257 genes upregulated and 793 downregulated (Figure 8B, Supplementary Table S4).

To investigate biological pathway dynamics, we performed KEGG enrichment analysis, which revealed that DEGs were predominantly enriched in cellular processes, environmental information processing, metabolism, and organismal systems. Most of the DEGs were involved in metabolic pathways. Interestingly, we found four down-regulated *TaABCB* genes (*TaABCB7*, *TaABCB16*, *TaABCB31*, *TaABCB50*) associated with environmental information processing (Figure 8C, Supplementary Table S4). GO enrichment analysis was subsequently performed to functionally annotate DEGs into two major categories: biological process (BP) and molecular function (MF) (Figure S1, Supplementary Table S4). This analysis identified 39 highly enriched GO terms under BP and 11 under MF. Notably, the GO term GO:0140359 (ABC-type transporter activity) within the MF category was enriched. Specifically, 5 *TaABCB* members (*TaABCB7, TaABCB16, TaABCB31, TaABCB35*, *TaABCB50*) were functionally associated with this functional annotation. This finding underscores their critical roles in phosphate starvation response, suggesting potential involvement in nutrient transport and metabolic adaptation under stress conditions.

### 3.7. Expression Profile of TaABCB Members in Response to Phosphate Starvation and the Analysis of Candidate Gene

**Figure 9:**
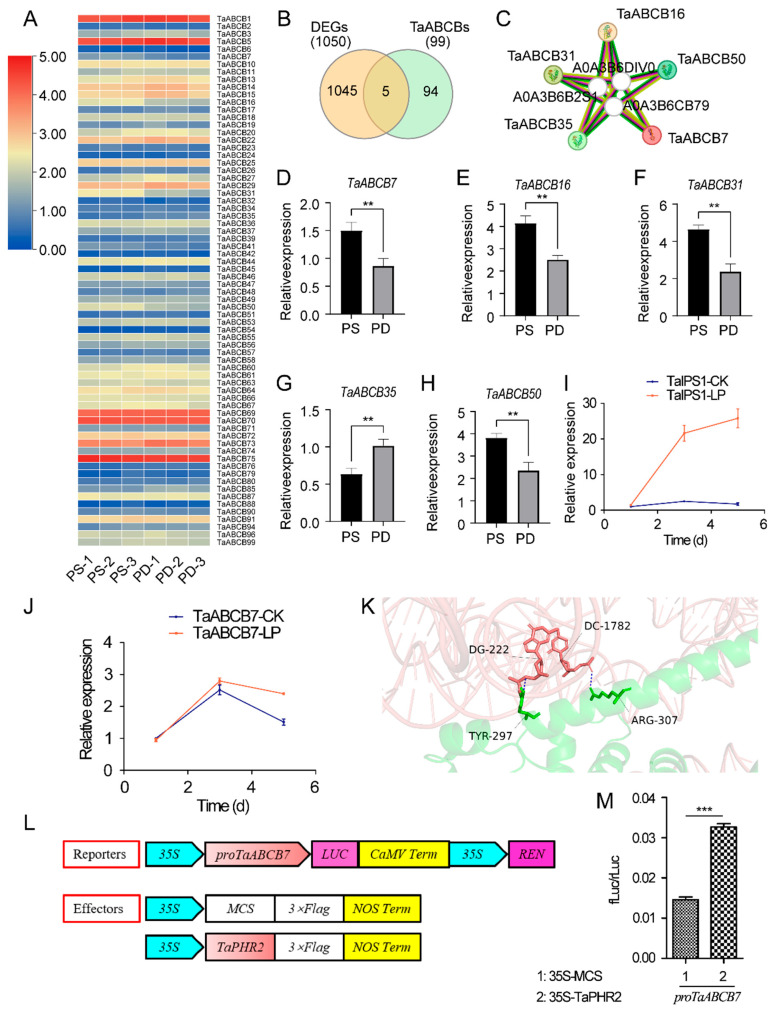
Expression profiles and candidate gene correlation analysis of *TaABCB* members. (**A**) Expression profiles of *TaABCB* members under low phosphorus conditions. (**B**) Transcriptome differentially expressed genes and Venn diagram between *TaABCB* members. (**C**) Prediction of protein interactions between differentially expressed genes in *TaABCB* members. (**D**–**H**) Expression analysis of candidate genes in *TaABCB* members under normal and low phosphorus conditions. (**I**,**J**) Under low phosphorus stress treatment conditions, the expression profiles of *TaIPS1* and *TaABCB7* genes at different time points. (**K**) AlphaFold 3 predicted the conjunction of TaPHR2 and the promoter of *TaABCB7*. (**L**,**M**) Dual-Luciferase reporter assays. Student’s *t*-test was performed to analyze the significance between PS and PD, ** *p*-value ≤ 0.01, and *** *p*-value ≤ 0.001.

To understand the expression profile of the *TaABCB* members in phosphorus starvation response, we visualized the expression of *TaABCB* members using phosphorus starvation transcriptome data (Figure 9A). Through colocalization of differentially expressed genes and *TaABCB* members in transcriptome data, five differentially expressed genes were identified (Figure 9B), indicating that these candidate genes are functionally involved in the phosphorus pathway. By using the STRING online tool to predict protein interactions, it was found that there is a certain degree interaction of differentially expressed genes (Figure 9C, Supplementary Table S5). Our transcriptomic analysis under low phosphorus conditions showed that four candidate genes were repressed while one was induced. Among these, TaABCB7 displayed a significantly higher expression fold change during phosphorus starvation versus Pi-replete conditions, indicating its potential involvement in phosphorus homeostasis (Figure 9D–H).

To further validate the accuracy of transcriptome results and identify key candidate genes, we analyzed *TaABCB7* expression under low phosphorus (LP) stress across multiple time points and further validated our findings using the LP stress marker gene *TaIPS1*. RT-qPCR analysis revealed that *TaIPS1* expression was markedly induced under LP stress compared to normal phosphorus levels, showing a time-dependent accumulation pattern (Figure 9I). These results align with prior studies, supporting our experimental conclusions. Interestingly, *TaABCB7* expression exhibited a slight downregulation after 1 day of LP stress relative to controls. But with the increase in stress time, compared to normal conditions, the expression level of *TaABCB7* gradually upregulated, and the upregulation amplitude gradually increased. At 3 days, the expression level reached its peak (Figure 9J). Based on the above results, it is suggested that *TaABCB7* may act as a key candidate gene which plays a more important role in participating in the phosphorus pathway.

Therefore, AlphaFold 3 was employed for predicting the conjunction between the transcription factor TaPHR2 and the promoter region of *TaABCB7*, while HDOCK was leveraged for conducting a docking analysis of the TaPHR2-*TaABCB7* interaction. The pTM + ipTM value, as computed by AlphaFold3 for both proteins and nucleic acids, amounted to 0.54, falling below the threshold of 0.75. Upon inspecting the AlphaFold3 model, it became evident that extensive flexible zones are present within both protein and nucleic acid components, accounting for the resultant low ipTM value. The docking score from HDOCK revealed an optimal conformation with a binding energy of −328.16 kcal/mol (surpassing the threshold of −200 kcal/mol) and a confidence score exceeding 0.7, indicating that a strong binding ability between TaPHR2 and the promoter of *TaABCB7*. Subsequently, the optimal conformation was visualized using Pymol software (version 3.1.0) to analysis the binding interface between the TaPHR2 and the promoter of *TaABCB7*. At this interface, hydrogen bonding interactions were identified between the 222nd guanine deoxyribonucleotide (dG) of the *TaABCB7* promoter and the 297th tyrosine residue (Tyr297) of the TaPHR2, as well as between the 1782nd cytosine deoxyribonucleotide (dC) of the *TaABCB7* promoter and the 307th arginine residue (Arg307) of the TaPHR2. These molecular interactions may serve as key determinants in stabilizing the complex structure (Figure 9K).

To further elucidate the transcriptional regulatory relationship between *TaABCB7* and *TaPHR2*, we conducted Dual-Luciferase reporter assays in protoplasts derived from *Nicotiana benthamiana* leaves (Figure 9L). As expected, *TaABCB7* expression was significantly enhanced when cotrasfommed with TaPHR2, which was drastically higher than those of the negative controls (Figure 9M). These results indicate that TaPHR2 as a transcriptional activator of *TaABCB7* could modulate phosphate starvation responses in wheat.

### 3.8. Haplotype Analysis of TaABCB7

**Figure 10:**
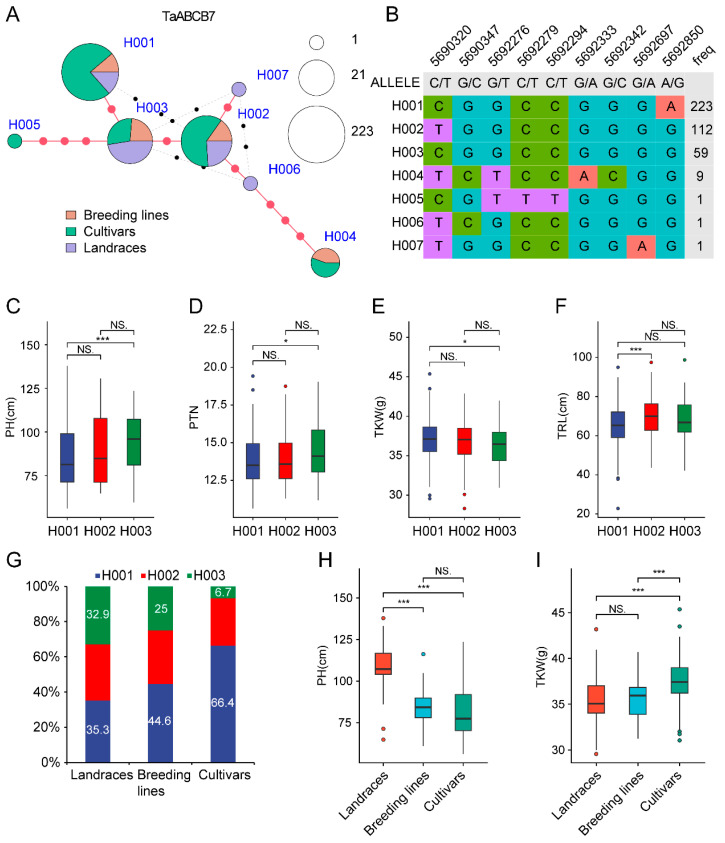
Haplotype analysis of the candidate gene *TaABCB7*. (**A**) Haplotype network diagram of *TaABCB7* in 406 wheat germplasms. H001-H007 represent different haplotypes, the size of the circle represents the amount of material contained in each haplotype, and different colors represent different varieties. (**B**) Table of haplotypes of *TaABCB7* in 406 wheat germplasms. The top number represents different SNP markers, and the right number represents the number of material portions. (**C**) Significance analysis of different haplotypes in plant height (PH). (**D**) Significance analysis of different haplotypes in productive tiller number (PTN). (**E**) Significance analysis of different haplotypes in thousand kernel weight (TKW). (**F**) Significance analysis of different haplotypes in total root length (TRL). (**G**) The distribution of different haplotypes in the process of breeding improvement. (**H**) Significance analysis of the elite haplotype H001 in plant height among different subgroup materials. (**I**) Significance analysis of the elite haplotype H001 in thousand kernel weight among different subgroup materials. Student’s *t*-test was performed to analyze the significance between different haplotypes, ns, no significant difference, * represents *p*-value ≤ 0.05, and *** *p*-value ≤ 0.001.

To further understand the selection of *TaABCB7* in the wheat breeding process, we extracted SNP markers of *TaABCB7* gene from 406 wheat germplasms. The results showed that there were seven haplotypes, with H001 haplotype containing 223 wheat germplasms, H002 haplotype containing 112 wheat germplasms, H003 haplotype containing 59 wheat germplasms, H004 haplotype containing 9 wheat germplasms, and all other haplotypes containing 1 wheat germplasm. There was little genetic difference between H001, H002, and H003 (Figure 10A,B). To meet the requirements of data statistics, we mainly selected H001, H002, and H003 haplotypes for subsequent analysis, and excluded the remaining haplotypes with few materials. Subsequently, significant analysis was conducted on plant height (PH), productive tiller number (PTN), thousand kernel weight (TKW) and total root length (TRL) among different haplotypes. The results showed that H002 did not exhibit significant differences when compared to H001 or H003, but H001 and H003 show significant differences in three traits (PH, PTN and TKW). There were significant differences in RL traits between H001 and H002. Wheat varieties with H001 show lower plant height, fewer tiller number, higher thousand kernel weight and shorter root length, indicating that H001 is an elite haplotype (Figure 10C–E). Interestingly, we found that the proportion of H001 gradually increased during the breeding improvement process, from 35.3% in local varieties to 66.4% in modern cultivated varieties, while the proportion of H003 gradually decreased, from 32.9% in local varieties to 6.7% in modern cultivated varieties, indicating that the elite haplotype H001 was selected during the breeding improvement process (Figure 10F). Furthermore, we observed that during the improvement process from local varieties to modern cultivated varieties, PH was significantly decreased, while TKW significantly increased (Figure 10H,I).

## 4. Discussion

The ABCB transporters are important multiple functional proteins mainly involved in material transport and metabolic regulation, and have profound effects on plant developmental processes and environmental adaptation. Therefore, the related research on ABCB transporters is of great significance.

### 4.1. The Characteristics of TaABCB Members in Wheat

To date, extensive research has identified and characterized numerous *ABCB* gene family members across multiple plant species, including *Arabidopsis thaliana*, rice, and maize. There are 29 *AtABCB* members in *Arabidopsis thaliana*, of which 22 multidrug resistance (MDR) proteins are full molecular transporters, while the rest are semi molecular transporters, including three ABC transporters of the mitochondria (ATM), three transporters associated with antigen processing (TAP), and one prokaryotic lipid A-like exporters (LLP) [50]. The rice genome contains 27 confirmed members of the *OsABCB* gene family, and the number of MDR, TAP and LLP is the same as that of *Arabidopsis thaliana*, with only one ATM. The *ABCB* subfamily of maize consists of 31 members, including 18 full molecular transporters, 12 half molecular transporters, and 1 member encoding an incomplete NBD (*ZmABCB19*) [51].

Through genome-wide mining, we identified 99 *TaABCB* genes in common wheat, representing a notable increase compared to *Arabidopsis thaliana*, rice, and maize. This gene family expansion is likely attributed to wheat’s hexaploid genome structure, which facilitates both whole-genome duplication-driven proliferation and segmental duplication-mediated amplification of *ABCB* genes. Subsequently, we further analyzed the physicochemical properties of all identified TaABCB proteins. Subcellular localization showed that the *TaABCB* family is located on the cell membrane, indicating its important role in signal perception and transmission. We used *AtABCB* subgroups as a reference [52], *TaABCB* members were divided into four large evolutionary branches, including 78 MDR type transporters, 6 ATM type transporters, 12 TAP type transporters and 3 LLP type transporters, indicating that genes encoding proteins with identical structural features tend to cluster together and may exhibit similarities in their functional roles. The different subgroups have different ABCB protein structural types, indicating functional differentiation of the *TaABCB* members during evolution. The investigation of conserved motifs and gene structures uncovered that members within the same subgroups exhibit similar motif distributions and gene architectures, whereas distinct differences exist between subgroups. These findings align with the constructed evolutionary tree.

Gene duplication events, particularly segmental and tandem duplications, play pivotal roles in gene family expansion and functional diversification. To further investigate the distribution and evolutionary mechanism of the *TaABCB* members, we conducted chromosome localization and collinearity analysis. Our results revealed an uneven distribution of TaABCB members across 21 wheat chromosomes, with 79 segmental duplication pairs identified but no tandem duplications. Interestingly, we found that these fragment duplication phenomena mainly occur on the same chromosome of the A, B, and D genomes, while only 9 pairs of fragment duplication genes were found between 1A, 1B, 1D and 3A, 3B, 3D. These findings imply that segmental duplication during wheat chromosome polyploidization was the dominant driver of *TaABCB* family expansion, accompanied by purifying selection. Meanwhile, synteny comparisons with *Arabidopsis thaliana*, rice, and maize revealed stronger collinearity between wheat and the grass species (rice/maize), supporting their closer phylogenetic relationship.

### 4.2. The Function and Regulatory Mechanism of TaABCB Members in Wheat

*ABCB* transporters, a highly conserved protein family in plants, participate in critical physiological pathways, including auxin and brassinosteroid transport, phototaxis, geotropism, organ development, and heavy metal transport in *Arabidopsis thaliana*. Previous studies have reported that an MDR type ABCB transporter, TaMDR1, is upregulated by aluminum toxicity and calcium flux inhibition in wheat [53]. Despite these findings, systematic functional characterizations of other *TaABCB* subfamily genes in wheat are still rare.

The composition of *cis*-acting elements within promoter regions is critical for determining gene regulatory networks and functional pathways. Our analysis identified numerous *cis*-acting elements in *TaABCB* promoters, with enrichment in motifs linked to plant development, phytohormone responses, light perception, and stress tolerance. These findings imply that *TaABCB* members may be regulated by different pathways, thereby involved in different pathways. For example, *AtABCB21* can modulate auxin distribution in *Arabidopsis thaliana* and play an important role in auxin transport [19], while *AtABCB19* overexpression alters hypocotyl elongation under red/blue light [54] and promotes the development of adventitious roots [55,56]. Additionally, *AtABCB4* can affect the development of lateral roots and root hair formation [57], and *OsABCB6* is induced under salt stress conditions [58]. Promoter analysis identified the presence of MYB-binding *cis*-elements in the regulatory regions of *TaABCB* genes, and MYB transcription factor, AtPHR1 or OsPHR2, is the core regulatory factors that regulate Pi starvation induction (PSI) gene. Therefore, we intend to explore the potential roles of *TaABCB* members in phosphate starvation response by RNA-seq analysis. Using phosphorus starvation transcriptome data and *TaABCB* gene family colocalization, five candidate differentially expressed genes were identified. STRING-based protein interaction predictions revealed protein–protein interactions among them, corroborating similar observations reported by McFarlane et al. [59]. The *TaABCB7* gene showed the most significant differential expression under phosphorus starvation conditions, indicating that it is a key candidate gene in participating in phosphorus starvation response. AlphaFold3 analysis suggests a potential binding ability of TaPHR2 to the promoter region of TaABCB7, and Dual-Luciferase reporter assays revealed that TaPHR2 could promote the expression of *TaABCB7*. These results indicate that TaPHR2 likely functions as a transcriptional activator of *TaABCB7*, plays a key role in the adaptation to low phosphorus availability by potentially modifying nutrient transport mechanisms.

### 4.3. The Haplotype of TaABCB7 in Wheat

As a globally vital food crop, wheat significantly contributes to global food security through enhanced productivity [60,61,62]. Identifying the elite haplotype of *TaABCB7* could improve the genetic improvement efficiency. Haplotype analysis revealed that the high allele frequency elite haplotype H001 with the characteristics of lower plant height, fewer effective tiller numbers, higher thousand kernel weight and shorter root length, was subjected to natural selection and utilized during the breeding and improvement process. The proportion of H003 gradually diminishes during the transition from local planting practices to modern breeding techniques. There is a notable decrease in plant height accompanied by a significant increase in TKW. This indicated that breeders prioritize selecting varieties with lower plant height and higher thousand grain weight, and the decrease in effective tiller number of the haplotype H001 may be due to an antagonistic relationship between this trait and the thousand grain weight trait. Although the elite haplotype H001 has been utilized by breeders in the improvement process, further enhancement of its application in future breeding is still necessary. At the same time, attention should be paid to the aggregation of this superior haplotype with other favorable traits, in order to construct ideal plant types and increase yield.

## 5. Conclusions

This study conducted a systematic investigation and identified 99 *TaABCB* gene family members in wheat, which were phylogenetically classified into four distinct branches (MDR, LLP, ATM, and TAP). These *TaABCB* genes exhibit functional diversity and are unevenly distributed on 21 chromosomes, with their expansion primarily driven by segmental duplication events. Transcriptomic profiling under phosphate starvation conditions uncovered five differentially expressed *TaABCB* genes, with *TaABCB7* as a key candidate gene and its expression is regulated by TaPHR2. Haplotype analysis of *TaABCB7* identified an elite haplotype (H001) linked to improved agronomic traits. Overall, these findings advance our understanding of *TaABCB* gene family and offer valuable genetic resources for breeding phosphate-efficient wheat cultivars.

## Data Availability

Data are contained within the article. The datasets presented in this study can be found in online repositories. The names of the repository/repositories and accession number(s) can be found below: https://submit.ncbi.nlm.nih.gov/subs/sra/ (accessed on 3 September 2025), PRJNA1314374.

## Supplementary Materials

The following supporting information can be downloaded at: https://www.mdpi.com/article/10.3390/biology14111525/s1, Figure S1: GO enrichment analysis of DEGs. Table S1: *AtABCB* and *OsABCB* transporters. Table S2: Physicochemical properties of *TaABCB* members. Table S3: KaKs analysis of *TaABCB* members. Table S4: Transcriptome sequencing data. Table S5: Prediction of protein interactions. Table S6: Primer sequences used in this study.
