# Supplementary figures and images for: Comprehensive Analysis of the TaABCB Gene Family and the Role of TaABCB7 in the Phosphate Starvation Response in Wheat
Source: Biology (Basel). 2025 Oct 30;14(11):1525. doi: 10.3390/biology14111525 (PMC12650226; doi:10.3390/biology14111525)

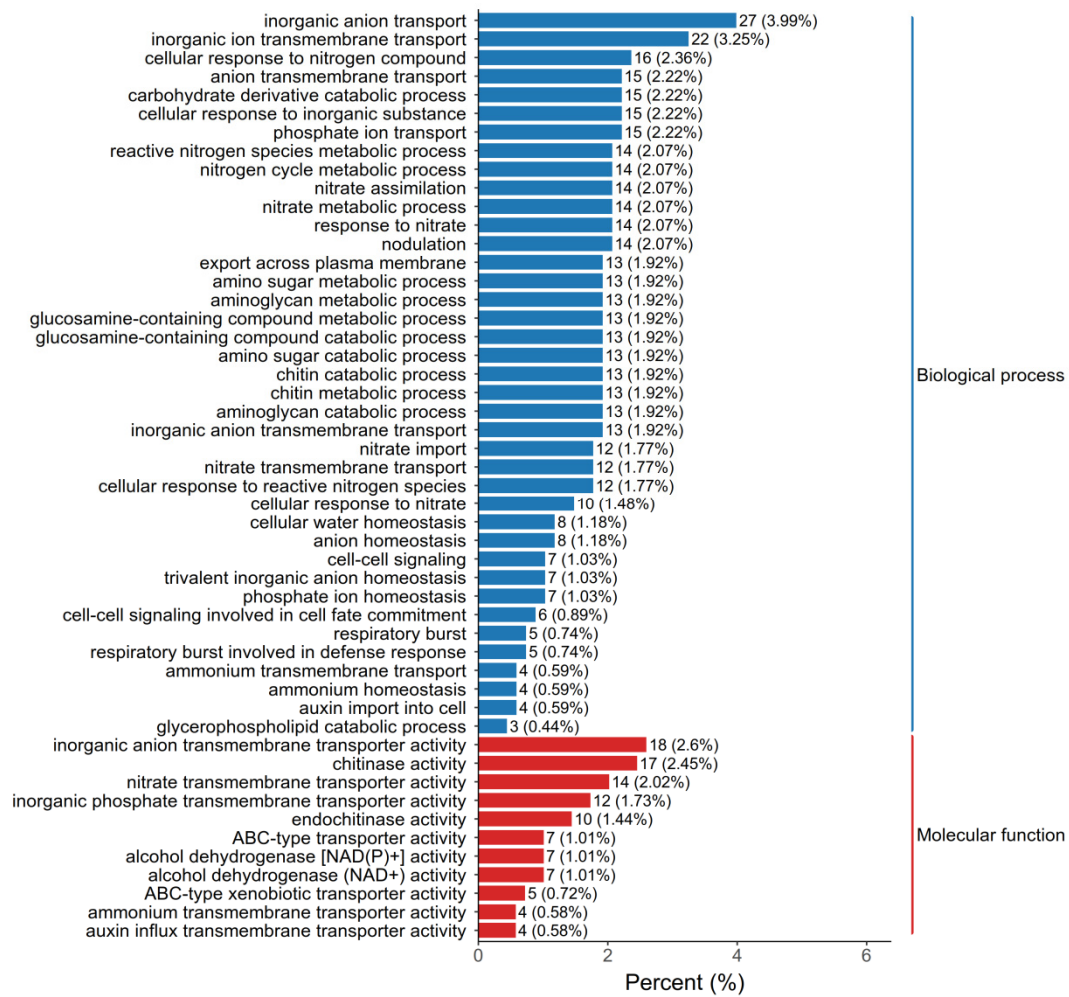

**Supplementary Figure S1 GO enrichment analysis of DEGs**

Supplement: Supplementary file 1 [file biology-14-01525-s001.zip › Supplementary Figure S1 GO enrichment analysis of DEGs.pdf]
